# Shock index to predict outcomes in patients with trauma following traffic collisions: a retrospective cohort study

**DOI:** 10.1007/s00068-024-02545-4

**Published:** 2024-05-31

**Authors:** Te-Kai Liao, Chung-Han Ho, Ying-Jia Lin, Li-Chin Cheng, Hsuan-Yi Huang

**Affiliations:** 1 Division of Traumatology, Department of Surgery, Chi Mei Medical Center, No. 901, Zhonghua Road, Yongkang District, 710 Tainan, Taiwan; 2 Department of Medicine Research, Chi Mei Medical Center, Tainan, Taiwan; 3 Department of Information Management, Southern Taiwan University of Science and Technology, Tainan, Taiwan; 4 Division of Colorectal Surgery, Department of Surgery, Chi Mei Medical Center, Tainan, Taiwan; 5 Center of General Education, Chia Nan University of Pharmacy and Science, Tainan, Taiwan

**Keywords:** Patient outcomes, Shock index, Traffic collision, Youden index

## Abstract

**Purpose:**

Taiwan, which has a rate of high vehicle ownership, faces significant challenges in managing trauma caused by traffic collisions. In Taiwan, traffic collisions contribute significantly to morbidity and mortality, with a high incidence of severe bleeding trauma. The shock index (SI) and the modified shock index (MSI) have been proposed as early indicators of hemodynamic instability. In this study, we aimed to assess the efficacy of SI and MSI in predicting adverse outcomes in patients with trauma following traffic collisions.

**Methods:**

This retrospective cohort study was conducted at Chi Mei Hospital from January 2015 to December 2020. The comprehensive analysis included 662 patients, with data collected on vital signs and outcomes such as mortality, blood transfusion, emergent surgical intervention (ESI), transarterial embolization (TAE), and intensive care unit (ICU) admission. Optimal cutoff points for SI and MSI were identified by calculating the Youden index. Logistic regression analysis was used to assess outcomes, adjusting for demographic and injury severity variables.

**Results:**

An SI threshold of 1.11 was associated with an increased risk of mortality, while an SI of 0.84 predicted the need for blood transfusion in the context of traffic collisions. Both SI and MSI demonstrated high predictive power for mortality and blood transfusion, with acceptable accuracy for TAE, ESI, and ICU admission. Logistic regression analyses confirmed the independence of SI and MSI as risk factors for adverse outcomes, thus, providing valuable insights into their clinical utility.

**Conclusions:**

SI and MSI are valuable tools for predicting mortality and blood transfusion needs in patients with trauma due to traffic collisions. These findings advance the quality of care for patients with trauma during their transition from the emergency room to the ICU, facilitating prompt and reliable decision-making processes and improving the care of patients with trauma.

## Background

In relation with the overall population of Taiwan, there is an almost equivalent number of vehicles and motorbikes, with 22,264,293 registered vehicles and motorbikes according to the Taiwan Ministry of Transportation and Communication [1]. The Taiwan National Police Agency (NPA) data for the year 2022 revealed that 375,632 traffic accidents resulted in injuries, including deaths that occurred immediately or within 24 h. In total, an alarming number of 3,085 people died as a result of traffic accidents, marking a 3.4% increase from the corresponding number in 2021 [2].

Despite its occurrence in a minority of patients, significant post-trauma bleeding is a major factor contributing death and morbidity [3, 4]. Early identification of severe bleeding trauma in patients has historically posed challenges [5], but this is necessary, as it would facilitate the implementation of aggressive interventions, such as emergent surgery, angiography with embolization, and early activation of massive transfusion protocol (MTP). Prompt and efficient application of these treatment approaches in patients with substantial bleeding can significantly impact the treatment course.

Allgower and Burri introduced the concept of shock index (SI) in 1967 as a tool to detect hypovolemic shock in patients with trauma. SI is determined by the ratio of heart rate (HR) to the systolic blood pressure (SBP), with a normal range of 0.5–0.7 [6]. SI is an easy-to-use index of circulatory dynamics, requiring no specialized tools or knowledge. Demonstrating greater sensitivity than vital signs alone, SI can identify early bleeding. Large retrospective studies have confirmed the predictive capacity of SI for hypotension, large transfusion need, and hypotension risk after intubation [7]. According to a previous research, SI has also been associated with hospital stay length, intensive care unit (ICU) stay length, ventilator support duration, blood consumption, and mortality [8–10]. Furthermore, modified SI (MSI), which is calculated as the ratio of HR to the mean arterial pressure (MAP), has also been proposed as a tool for assessing hemodynamic stability and has proven to be a more accurate indicator of mortality than the traditional SI for emergency patients [11].

However, most studies tend to aggregate all trauma mechanisms without specifically focusing on traffic accidents. In this study, we aimed to assess the validity of SI and MSI in predicting mortality, blood transfusion need, emergent surgical intervention (ESI), transarterial embolization (TAE), and ICU hospitalization in patients with trauma involved in traffic collisions at a Level 1 trauma center due to the high incidence of these events.

## Methods

### Study design and patient population

In this retrospective cohort study, we examined patients with trauma caused by traffic collision who were admitted to the emergency department (ED) of Chi Mei Hospital from January 2015 to December 2020. Chi Mei Hospital is a Level 1 facility with > 100 adult ICU beds and 800 regular beds, serving as a crucial reference point for trauma in Southern Taiwan. The hospital admits approximately 3,000 adult patients with trauma annually. With 3,226 casualties, the hospital is the principal trauma center for > 2 million residents of the city, which has the highest occurrence rate per 100,000 people in the nation.

### Inclusion and exclusion criteria

**Fig. 1:**
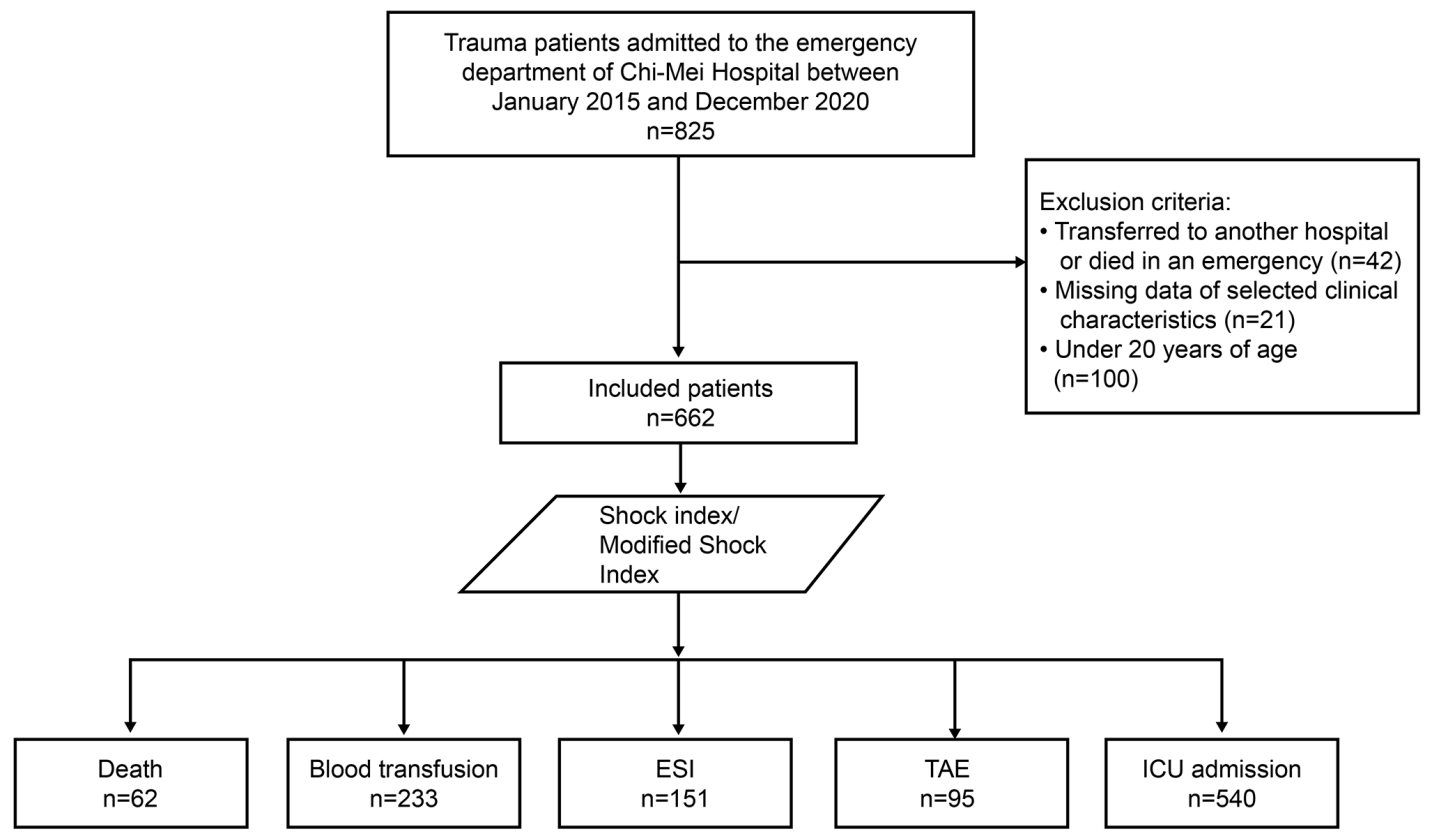
Flowchart depicting patient inclusion/exclusion criteria

This study only included patients with trauma caused by traffic collisions, who were admitted to the hospital and had full datasets for SBP, diastolic blood pressure (DBP), and HR obtained at the ED for SI and MSI calculation. Patients aged < 20 years were excluded from the study based on hospital ethical guidelines (Fig. 1).

### Measurements and outcomes

Physical parameters were measured to calculate the SI and MSI (SI = HR/SBP; MSI = HR/MAP). The optimal cutoff points for SI and MSI with respect to all relevant outcomes were determined by calculating the Youden index. This index, calculated as the sum of the test sensitivity and specificity minus 1, is particularly valuable for comparing the predictive capabilities of different thresholds for the same diagnostic test. Mortality, blood transfusion, ESI, TAE, and ICU hospitalization data were also examined.

In our study, blood transfusion was defined as administration of more than four units of packed red blood cells (PRBCs) within the initial 24 h. ESI includes exploratory laparotomy and exploratory thoracotomy performed within the first 72 h of care, and TAE was performed within 72 h of treatment.

### Statistical analysis

The baseline characteristics of the study participants are expressed as means and standard deviations for continuous variables or as frequencies and percentages for categorical variables. Differences in distribution were estimated using Pearson’s chi-square test for categorical variables and Student’s t-test for continuous variables.

The risks for outcomes of interest were estimated using logistic regression analysis. Odds ratios (ORs) with 95% confidence intervals (CIs) were calculated after adjusting for sex, age, and injury severity scores (ISS). Receiver operating characteristic (ROC) curves were used to assess precision of the outcomes of interest in the SI and MSI models. Differences between two ROC curves were compared using the DeLong test [12].

All statistical analyses were performed using SAS software (version 9.4; SAS Institute, Inc., Cary, NC, USA). The level of significance was set at *P* < 0.05.

## Results

This study included a cohort of 662 patients, with an average age of 46.76 ± 18.60 years; 435 (65.71%) and 227 (34.29%) were aged < 60 and > 60 years, respectively. The majority of patients were men (63%). Most patients had significant injury severity (≥ 16) (*n* = 484, 73.11%), a criterion commonly employed retrospectively to determine the appropriate activation of a trauma team. and a length of hospital stay of > 14 days (*n* = 301, 45.47%). The mean SBP, DBP, and HR of the study participants are summarized in Table 1. The mean SI and MSI were calculated as 0.82 ± 0.38 and 1.09 ± 0.47, respectively (Table 1).

**Table 1:** Patient characteristics

Characteristics (*n* = 662)		*n* (%) or mean ± SD
Gender		
	Male	418 (63.14)
	Female	244 (36.86)
Age group (years)		
	20–39 years	227 (34.29)
	49–59 years	208 (31.42)
	≥ 60 years	227 (34.229)
Injury Severity		
	Mild (≤ 8)	43 (6.50)
	Moderate (9–15)	135 (20.39)
	Significant (≥ 16)	484 (73.11)
Length of hospital stay		
	1–7 days	155 (23.41)
	8–14 days	206 (31.12)
	> 14 days	301 (45.47)
Vitals		
Systolic blood pressure (mmHg)		127.45 ± 36.72
Diastolic blood pressure (mmHg)		77.79 ± 20.67
Heart rate (beats/minute)		94.02 ± 21.23
Shock index		0.82 ± 0.38
Modified shock index		1.09 ± 0.47

Abbreviation: SD: standard deviation

The distribution of the outcomes of interest are shown in Table 2. The blood transfusion and ESI rates were 35.20% (*n* = 233) and 22.81% (*n* = 151), respectively. The incidence of TAE was 14.35% (*n* = 95), the ICU admission rate was 81.57% (*n* = 540), and the mortality rate was 9.37% (*n* = 62).

**Table 2:** Outcomes of interest

Outcome		*n*	%
Death			
	Yes	62	9.37
	No	600	90.63
Blood transfusion			
	Yes (≥ 4 U)	233	35.20
	No (0 or < 4 U)	429	64.80
ESI			
	Yes	151	22.81
	No	511	77.19
TAE			
	Yes	95	14.35
	No	567	85.65
ICU admission			
	No	122	18.43
	1–7 days	339	51.21
	8–14 days	109	16.47
	> 14 days	92	13.90

Abbreviations: ESI: emergent surgical intervention; ICU: intensive care unit; TAE: transarterial embolization

Table 3 presents the cutoff points of SI or MSI for the different outcomes and the associated risks. The Youden index yielded an SI threshold cutoff point of 1.11 (normal range: 0.5–0.7) and an MSI threshold cutoff point of 1.12 for predicting mortality. When predicting the need for blood transfusion, the threshold cutoff point was 0.84 for SI and 1.11 for MSI. To predict the requirement for ESI, the threshold cutoff point was 0.95 for SI and 1.18 for MSI. For predicting vascular thrombosis, the SI threshold cutoff point was 0.66 and MSI threshold cutoff point was 1.11. Finally, for ICU admission, the SI and MSI threshold cutoff points were 0.74 and 1.01, respectively. Logistic regression analysis, adjusting for age, sex, ISS, SI, and MSI, confirmed that an SI of 1.11 (OR, 6.38; 95%, CI, 3.39 − 12.03; *P* < 0.0001) and MSI of 1.12 (OR, 4.44; 95%, CI, 2.47 − 7.98; *P* < 0.0001) were independent predictors of mortality risk. In addition, an SI of 0.84 (OR, 9.60; 95% CI, 6.36 − 14.50; *P* < 0.0001) and MSI of 1.11 (OR, 10.52; 95% CI, 6.93 − 15.97; *P* < 0.0001) were independent risk factors for blood transfusion. For ESI, an SI of 0.95 (OR, 2.89; 95% CI, 1.92 − 4.35; *P* < 0.0001) and MSI of 1.18 (OR, 2.64; 95% CI, 1.78 − 3.94; *P* < 0.0001) were independent prognostic risk factors for emergent operation. An SI of 0.66 (OR, 3.14; 95% CI, 1.80 − 5.46; *P* < 0.0001) and MSI of 1.11 (OR, 2.43; 95% CI, 1.54 − 3.84; *P* = 0.0001) were independent prognostic factors of risk for TAE. Additionally, an SI of 0.74 (OR, 4.52; 95% CI, 2.69 − 7.61; <0.0001) and MSI of 1.01 (OR, 5.22; 95% CI, 3.02 − 9.03; <0.0001) were independent risk factors for ICU admission. In the regression analysis, only ISS ≥ 16 was identified as a significant independent predictor for both the need of blood transfusion and the duration of an ICU stay.

**Table 3:** Associated risk of each outcome for SI or MSI

	Crude OR	*P*-value	Adjusted OR^#^	*P*-value
Mortality				
SI (ref: ≤1.11)	4.98 (2.85, 8.69)	< 0.0001	6.38 (3.39, 12.03)	< 0.0001
MSI (ref: ≤1.12)	3.37 (1.98, 5.75)	< 0.0001	4.44 (2.47, 7.98)	< 0.0001
Blood transfusion				
SI (ref: ≤0.84)	8.81 (6.11, 12.71)	< 0.0001	9.60 (6.36, 14.50)	< 0.0001
MSI (ref: ≤1.11)	9.40 (6.49, 13.61)	< 0.0001	10.52 (6.93, 15.97)	< 0.0001
ESI				
SI (ref: ≤0.95)	3.10 (2.10, 4.58)	< 0.0001	2.89 (1.92, 4.35)	< 0.0001
MSI (ref: ≤1.18)	2.87 (1.96, 4.20)	< 0.0001	2.64 (1.78, 3.94)	< 0.0001
TAE				
SI (ref: ≤0.66)	3.32 (1.92, 5.72)	< 0.0001	3.14 (1.80, 5.46)	< 0.0001
MSI (ref: ≤1.11)	2.61 (1.68, 4.05)	< 0.0001	2.43 (1.54, 3.84)	0.0001
ICU admission				
SI (ref: ≤0.74)	4.95 (3.04, 8.07)	< 0.0001	4.52 (2.69, 7.61)	< 0.0001
MSI (ref: ≤1.01)	5.55 (3.32, 9.27)	< 0.0001	5.22 (3.02, 9.03)	< 0.0001

^#^adjusted for sex, age group, ISS group

Abbreviations: ESI: emergent surgical intervention; ICU: intensive care unit; ISS: injury severity score; MSI: modified shock index; OR: odds ratio; SI: shock index; TAE: transarterial embolization

**Fig. 2:**
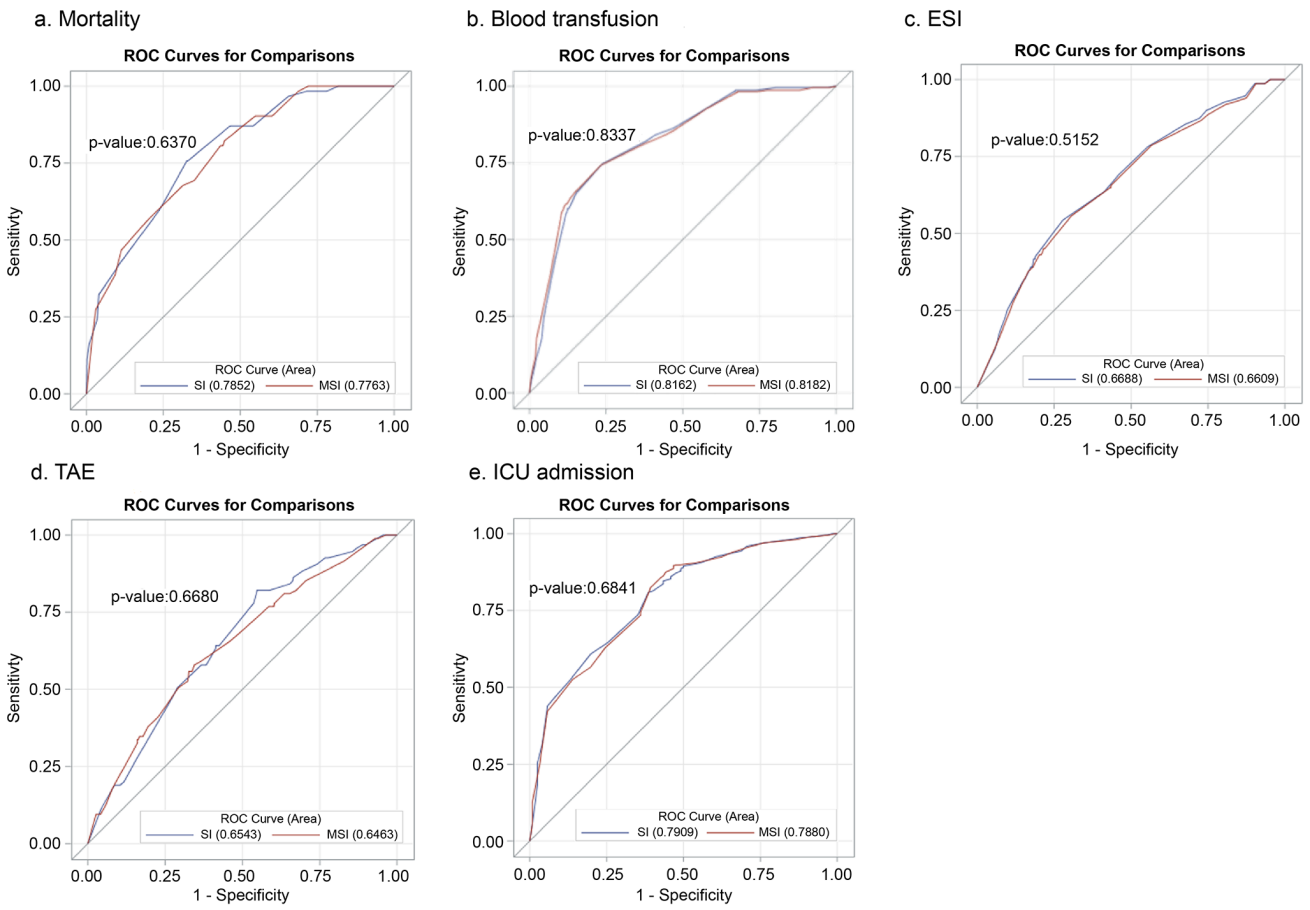
ROC curves for different outcomes in terms of the SI and MSI models (**a**) mortality, (**b**) blood transfusion, (**c**) ESI, (**d**) TAE, and (**e**) ICU admission outcomes The *p*-values are calculated by comparing the AUC between SI and MSI. Abbreviations: AUC: area under the curve; ESI: emergent surgical intervention; ICU: intensive care unit; MSI: modified shock index; ROC: receiver operating characteristic; SI: shock index; TAE: transarterial embolization

Comparison of the area under the ROC curve (AUROC) of SI and MSI for predicting mortality, blood transfusion, ESI, TAE, and ICU admission (Fig. 2) revealed that both SI and MSI showed high predictive power for mortality (AUROC = 0.7852 and 0.7763, respectively; *P* = 0.6370) and blood transfusion (AUROC = 0.8162 and 0.8182, respectively; *P* = 0.8337). Additionally, both SI and MSI showed lower, but acceptable, predictive power for TAE (AUROC = 0.6543 and 0.6436, respectively; *P* = 0.6680), ESI (AUROC = 0.6688 and 0.6609, respectively; *P* = 0.5152), and ICU admission (AUROC = 0.7909 and 0.7880, respectively; *P* = 0.6841). The predictive power did not differ between SI and MSI in any of the outcomes, including mortality, blood transfusion, ESI, TAE, and ICU admission.

## Discussion

To the best of our knowledge, this study is the first to apply SI and MSI in the context of injury mechanisms resulting from traffic collisions.

Berger et al. found that SI ≥ 1.0 exhibited the highest specificity as a predictor for both 28-day mortality and hyperlactatemia in cases of sepsis presenting to the ED [13]. Dissimilar from our results, Montoya et al. found that mortality was correlated with an SI > 0.9 in patients with acute polytrauma [14]. However, their study included both penetrating and blunt injuries, which differed significantly from the injuries included in the present study. We realize that different groups of patients will have different reference values when using SI and that it is highly valuable to allow frontline healthcare providers to assess the severity of conditions and be more vigilant in caring for different patients [13, 15, 16]. Our study population was derived from traffic collisions, in which all patients had blunt injury mechanisms. Therefore, mortality outcomes correlated with an SI > 1.11 may only be particularly applicable to this population.

In this study, SI > 0.84 upon arrival in the initial level of care for individuals involved in traffic collisions serves as a predictor for the need of blood transfusion, as determined by the Youden index. A similar approach to the SI cutoff value was reported by Marenco et al. [17], wherein they investigated significant factors associated with the requirement for massive transfusion (MT) and emergency surgery in patients with trauma within the civilian population, identifying an SI cutoff point of 0.8. In a previous retrospective analysis involving 8111 civilian patients with blunt trauma admitted to a Level 1 trauma center, SI > 0.9 was correlated with a substantially elevated likelihood of requiring MT [18]. In a recent retrospective study by El-Menyar et al., which included 8710 civilian patients with trauma admitted to a Level 1 trauma center, SI > 0.8 on arrival emerged as a significant predictor for MTP, requirement for laparotomy, and in-hospital mortality [19].

Through a comprehensive review of existing studies, we defined the need for blood transfusion as administration of four units of PRBCs within 24 h. This rationale behind this definition was partly influenced by our hospital’s transfusion practices, which align closely with the onset of changes in vital signs in grade II hemorrhagic shock, indicating the need for blood transfusion. In other studies, such as those by DeMuro et al., bleeding was defined as the receipt of two units of PRBCs [20]. Conversely, many studies have defined MT as administration of 10 units of PRBCs [17, 21]. We adopted an intermediate approach, setting the threshold at 4 PRBC units. With a Youden index falling between 0.8 and 0.9 and an OR over nine-fold, we can confidently assert that SI > 0.84 predicts the need for blood transfusion.

Crawford et al. reported that with each 0.1 unit rise in SI, the odds of requiring ESI increased by 21% in surgical patients. The SI was higher for blunt injuries than for penetrating injuries (0.95 vs. 0.73) [22]. Given that our patient population comprised exclusively of blunt trauma cases, our analysis aligned perfectly with the previous blunt trauma subgroup results, with the same value of 0.95. We can infer that penetrating injuries may cause more noticeable wounds, leading to a higher likelihood of surgery, even before hemodynamic changes occur. In contrast, blunt trauma may not exhibit obvious signs of bleeding or the need for surgical investigation of the open wound, prompting the need for surgery only after a certain degree of blood loss and the onset of hemodynamic changes, resulting in higher SI. We posit that employing SI at the bedside could prove invaluable in low-resource settings by providing early notification to clinicians regarding patients with initially stable vital signs but with a heightened probability of needing ESI or facing mortality.

Upon reviewing the literature related to SI and TAE, studies primarily focused on cases with known severe localized bleeding, such as in the facial area. These studies used SI to assess the severity of bleeding but did not provide any direct data for using SI as a predictive tool for the necessity of TAE [23, 24]. In contrast, our results demonstrated acceptable predictive power for using SI to determine the need for TAE. However, the use of SI and MSI for TAE prediction should be subjected to further statistical analyses and the development of more precise research methodologies.

Finally, ICU admission was correlated with SI > 0.74 in our study. In comparison, SI ≥ 0.85 was indicative of ICU admission in a retrospective analysis conducted at a single center by Keller et al. [25]. Another study demonstrated that SI ≥ 0.9 upon initial arrival at the ED served as a predictor for both in-hospital mortality and ICU admission [26]. However, the study population included general patients in the ED, instead of patients with trauma. Toccaceli et al. found that an SI threshold value of 1.05 demonstrated the highest predictive efficacy for ICU admission in patients with multiple traumas [27]. Comparing our own results for patients involved in traffic collisions, the SI in these previous studies vary to some extent, possibly because of the different populations or different mechanisms of injury. Additionally, the results may have been influenced by our medical strategies and the relatively high availability of ICUs.

MAP best represents the tissue perfusion status, and MSI reflects both stroke volume and systemic vascular resistance [11]. MSI serves as a crucial predictor of mortality, surpassing the individual predictive capacity of HR, SBP, and SI, with an optimal cutoff value exceeding 1.3 [28]. In contrast, a multicenter study examining data from 45,880 patients with trauma across 20 EDs reported no discernible differences in the predictive capacity of SI and MSI for in-hospital mortality [29]. Herein, we aimed to investigate whether MSI was more accurate than SI in its predictive value and found that MSI did not exhibit superior discriminatory ability compared to SI in predicting outcomes, such as mortality, blood transfusion, ESI, TAE, or ICU stay in our cohort.

When managing cases involving traffic collisions, patients who are admitted to the ED are in a race against time to receive treatment. At our Level I trauma center, we noted a recurring occurrence wherein patients often experienced a substantial delay in receiving treatment, even after undergoing comprehensive evaluation and completing all requisite imaging and diagnostic procedures. In this context, SI and MSI are uncomplicated scoring systems suitable for bedside applications that can provide quick and reliable treatment decision-making to expedite this process.

This study had some limitations. First, because of the retrospective study design, there was a potential for selection bias, and our sample population, compared with that of various international studies [17–19, 22], was relatively small. Second, our hospital did not implement an MTP, instead relying on routine methods, such as transfusion guidelines. Therefore, the criteria for transfusion in our study were relatively lenient compared to those requiring MTP activation. Finally, we did not strictly define the type of surgery used; therefore, some surgeries that were not life-related may have been included in the data analysis, resulting in inaccurate results. In the future, as the dataset becomes more extensive, it will be feasible to categorize the surgical procedures into distinct types. Our data appear to be significant for a Taiwanese population and allows us to draw conclusions that apply to our local situation.

## Conclusions

Our investigation revealed that SI and MSI are precise and reliable indicators for predicting mortality and blood transfusion requirements in cases of trauma resulting from traffic collisions. We identified an upper threshold value for SI of 1.11 in predicting mortality and an SI of 0.84 in predicting the need for blood transfusion. The use of SI and MSI in predicting ESI, TAE, and ICU admission were also acceptable. These findings may contribute to improving the care and management of patients with trauma across both the ED and the ICU.

## Data Availability

All data generated or analyzed during this study are included in this published article.

## Abbreviations

AUROC: area under the receiver operating characteristic curve
CI: confidence interval
ED: emergent department
ESI: emergent surgical intervention
HR: heart rate
ICU: intensive care unit
ISS: injury severity score
MAP: mean arterial pressure
MSI: modified shock index
MT: massive transfusion
MTP: massive transfusion protocol
NPA: National Police Agency
OR: odds ratio
PRBCs: packed red blood cells
ROC: receiver operating characteristic
SBP: systolic blood pressure
SI: shock index
TAE: transarterial embolization

## References

[1] MOTC. The Statistical reports of Taiwan Ministry of Transportation and Communication. 2021. https://stat.motc.gov.tw/mocdb/stmain.jsp?sys=100&funid=a3301

[2] NPA. The annual statistics report of national police agency in 2022. https://www.npa.gov.tw/ch/app/data/view?module=wg056&id=2217&serno=fbf5a752-9c50-4eb1-a3ac-8d4f7112c70a

[3] Thomas M, Haas TS, Doerer JJ, Hodges JS, Aicher BO, Garberich RF, et al. Epidemiology of sudden death in young, competitive athletes due to blunt trauma. Pediatrics. 2011;128:e1–8. 21690117 10.1542/peds.2010-2743

[4] Como JJ, Dutton RP, Scalea TM, Edelman BB, Hess JR. Blood transfusion rates in the care of acute trauma. Transfusion. 2004;44:809–13. 15157244 10.1111/j.1537-2995.2004.03409.x

[5] Chico-Fernández M, García-Fuentes C, Alonso-Fernández MA, Toral-Vázquez D, Bermejo-Aznarez S. Alted-López E. [Massive transfusion predictive scores in trauma. Experience of a transfusion registry]. Med Intensiva. 2011;35:546–51. 21906847 10.1016/j.medin.2011.06.010

[6] Allgöwer M, Burri C. Shock-index. Ger Med Mon. 1968;13:14–9. 5656857

[7] Birkhahn RH, Gaeta TJ, Terry D, Bove JJ, Tloczkowski J. Shock index in diagnosing early acute hypovolemia. Am J Emerg Med. 2005;23:323–6. 15915406 10.1016/j.ajem.2005.02.029

[8] Grimme K, Pape HC, Probst C, Seelis M, Sott A, Harwood P, et al. Calculation of different triage scores based on the German trauma registry. Eur J Trauma. 2005;31:480–7.

[9] Cannon CM, Braxton CC, Kling-Smith M, Mahnken JD, Carlton E, Moncure M. Utility of the shock index in predicting mortality in traumatically injured patients. J Trauma. 2009;67:1426–30. 20009697 10.1097/TA.0b013e3181bbf728

[10] McNab A, Burns B, Bhullar I, Chesire D, Kerwin A. A prehospital shock index for trauma correlates with measures of hospital resource use and mortality. Surgery. 2012;152:473–6. 22938906 10.1016/j.surg.2012.07.010

[11] Liu YC, Liu JH, Fang ZA, Shan GL, Xu J, Qi ZW, et al. Modified shock index and mortality rate of emergency patients. World J Emerg Med. 2012;3:114–7. 25215048 10.5847/wjem.j.issn.1920-8642.2012.02.006 PMC4129788

[12] DeLong ER, DeLong DM, Clarke-Pearson DL. Comparing the areas under two or more correlated receiver operating characteristic curves: a nonparametric approach. Biometrics. 1988;44:837–45. 3203132

[13] Berger T, Green J, Horeczko T, Hagar Y, Garg N, Suarez A, et al. Shock index and early recognition of sepsis in the emergency department: pilot study. West J Emerg Med. 2013;14:168–74. 23599863 10.5811/westjem.2012.8.11546 PMC3628475

[14] Montoya KF, Charry JD, Calle-Toro JS, Núñez LR, Poveda G. Shock index as a mortality predictor in patients with acute polytrauma. J Acute Dis. 2015;4:202–4.

[15] Choi JY, Lee WH, Yoo TK, Park I, Kim DW. A new severity predicting index for hemorrhagic shock using lactate concentration and peripheral perfusion in a rat model. Shock. 2012;38:635–41. 23143055 10.1097/SHK.0b013e318273299f

[16] Bruijns SR, Guly HR, Bouamra O, Lecky F, Lee WA. The value of traditional vital signs, shock index, and age-based markers in predicting trauma mortality. J Trauma Acute Care Surg. 2013;74:1432–7. 23694869 10.1097/TA.0b013e31829246c7

[17] Marenco CW, Lammers DT, Morte KR, Bingham JR, Martin MJ, Eckert MJ. Shock index as a predictor of massive transfusion and emergency surgery on the modern battlefield. J Surg Res. 2020;256:112–8. 32683051 10.1016/j.jss.2020.06.024

[18] Vandromme MJ, Griffin RL, Kerby JD, McGwin G Jr., Rue LW 3rd, Weinberg JA. Identifying risk for massive transfusion in the relatively normotensive patient: utility of the prehospital shock index. J Trauma. 2011;70:384–8. 21307738 10.1097/TA.0b013e3182095a0a

[19] El-Menyar A, Goyal P, Tilley E, Latifi R. The clinical utility of shock index to predict the need for blood transfusion and outcomes in trauma. J Surg Res. 2018;227:52–9. 29804862 10.1016/j.jss.2018.02.013

[20] DeMuro JP, Simmons S, Jax J, Gianelli SM. Application of the shock index to the prediction of need for hemostasis intervention. Am J Emerg Med. 2013;31:1260–3. 23806728 10.1016/j.ajem.2013.05.027

[21] Carsetti A, Antolini R, Casarotta E, Damiani E, Gasparri F, Marini B, et al. Shock index as predictor of massive transfusion and mortality in patients with trauma: a systematic review and meta-analysis. Crit Care. 2023;27:85. 36872322 10.1186/s13054-023-04386-w PMC9985849

[22] Crawford R, Kruger D, Moeng M. Shock index as a prognosticator for emergent surgical intervention and mortality in trauma patients in Johannesburg: a retrospective cohort study. Ann Med Surg (Lond). 2021;69:102710. 34429962 10.1016/j.amsu.2021.102710 PMC8365323

[23] Kim J, Park SK, Chung J. Role of transarterial embolization in the treatment of life-threatening hemorrhage in patients with maxillofacial injury. Korean J Neurotrauma. 2022;18:178–87. 36381464 10.13004/kjnt.2022.18.e37 PMC9634291

[24] Hsu FY, Mao SH, Chuang AD-C, Wong YC, Chen CH. Shock index as a predictor for angiographic hemostasis in life-threatening traumatic oronasal bleeding. Int J Environ Res Public Health. 2021;18:11051. 34769572 10.3390/ijerph182111051 PMC8582879

[25] Keller AS, Kirkland LL, Rajasekaran SY, Cha S, Rady MY, Huddleston JM. Unplanned transfers to the intensive care unit: the role of the shock index. J Hosp Med. 2010;5:460–5. 20945470 10.1002/jhm.779

[26] Surendhar S, Jagadeesan S, Jagtap AB. Complementary value of the Shock Index v. the modified shock index in the prediction of in-hospital intensive care unit admission and mortality: a single-centre experience. Afr J Thorac Crit Care Med. 2023;29. 10.7196/AJTCCM.2023.v29i2.286 PMC10446160 37622103

[27] Toccaceli A, Giampaoletti A, Dignani L, Lucertini C, Petrucci C, Lancia L. The role of shock index as a predictor of multiple-trauma patients’ pathways. Nurs Crit Care. 2016;21:e12–9. 25641362 10.1111/nicc.12152

[28] Singh A, Ali S, Agarwal A, Srivastava RN. Correlation of shock index and modified shock index with the outcome of adult trauma patients: a prospective study of 9860 patients. N Am J Med Sci. 2014;6:450–2. 25317389 10.4103/1947-2714.141632 PMC4193151

[29] Kim SY, Hong KJ, Shin SD, Ro YS, Ahn KO, Kim YJ, et al. Validation of the shock index, modified shock index, and age shock index for predicting mortality of geriatric trauma patients in emergency departments. J Korean Med Sci. 2016;31:2026–32. 27822945 10.3346/jkms.2016.31.12.2026 PMC5102870

